# Comparison of Carotid Intima-Media Thickness in Pediatric Patients with Metabolic Syndrome, Heterozygous Familial Hyperlipidemia and Normals

**DOI:** 10.1155/2014/546863

**Published:** 2014-05-14

**Authors:** Arvind Vijayasarathi, Stanley J. Goldberg

**Affiliations:** ^1^Emory University Hospital Radiology, Atlanta, GA 30322, USA; ^2^University of Arizona College of Medicine, Tucson, AZ 85724, USA

## Abstract

*Background.* Our goal was to compare the carotid intimal-medial thickness (CIMT) of untreated pediatric patients with metabolic syndrome (MS), heterozygous familial hyperlipidemia (heFH), and MS+heFH against one another and against a control group consisting of healthy, normal body habitus children.* Methods.* Our population consisted of untreated pediatric patients (ages 5–20 yrs) who had CIMT measured in a standardized manner.* Results.* Our population included 57 with MS, 23 with heFH, and 10 with MS+heFH. The control group consisted of 84 children of the same age range. Mean CIMT for the MS group was 469.8 **μ**m (SD = 67), 443.8 **μ**m (SD = 61) for the heFH group, 478.3 **μ**m (SD = 70) for the MS+heFH group, and 423.2 **μ**m (SD = 45) for the normal control group. Significance differences between groups occurred for heFH versus MS (*P* = 0.022), heFH versus control (*P* = 0.038), MS versus control (*P* = 9.0E − 10), and MS+heFH versus control (*P* = 0.003). Analysis showed significant negative correlation between HDL and CIMT (*r* = −0.32, *P* = 0.03) but not for LDL, triglycerides, BP, waist circumference, or BMI.* Conclusion.* For pediatric patients, the thickest CIMT occurred for patients with MS alone or for those with MS+heFH. This indicates that MS, rather than just elevated LDL, accounts for more rapid thickening of CIMT in this population.

## 1. Introduction

Although clinical manifestations of atherosclerosis are observed mainly in the adult population, atherosclerosis has its beginning in the pediatric age range. The pathogenesis of early atherosclerosis as identified by the Pathobiological Determinants of Atherosclerosis in Youth (PDAY) trial begins with a fatty streak of lipid filled macrophages accumulating in the intima of an artery. Next, it was shown that vascular smooth muscle proliferated and entered the intima forming a fibrous area [1]. A common manifestation of early atherosclerosis in children and teenagers is diffuse thickening of the intima-media space rather than discrete lipid core and fibrous cap formation [2].

The Bogalusa Heart Trial (Bogalusa), a long term trial following patients from childhood to adult life, identified increased LDL, low HDL, elevated blood pressure, diabetes, and overweight/obesity as the most significant risk factors in the transition from pediatric to adult vascular disease [3]. These risk factors, well known in adult atherosclerosis, have been accepted by the American Academy of Pediatrics as risk factors in the pediatric age range [4]. The JUPITER study identified elevated high sensitivity C reactive protein (hsCRP) as an inflammatory biomarker that is independently associated with future cardiovascular events, even in adults with relatively low LDL levels [5].

Measurement of common carotid artery (CCA) intima-media thickness (CIMT) is clinically useful as an indicator of subclinical atherosclerosis and as predictive of cardiovascular events in the adult population [6–10]. Pediatric subgroups that have been shown to have increased CIMT include those with pediatric patients with chronic kidney disease [11], type I diabetes [12, 13], elevated high sensitivity CRP (hsCRP) [14], obesity [15, 16], type 2 diabetes [17], hypertension [18], and familial hyperlipidemia [19]. Even though it is now established that elevated LDL, non-HDL levels, and inflammatory biomarkers are associated with development of atherosclerosis, it is unclear which process results in earlier and more significant vascular changes in the pediatric age range.

The purpose of this study was to compare CIMT in pediatric patient groups with metabolic syndrome (MS), heterozygous familial hyperlipidemia (heFH) and in a group with both MS and heFH (MS+heFH) in order to determine which process results in more rapid thickening of CIMT.

## 2. Methodology

The Institutional Review Board of the University of Arizona approved the protocol for this retrospective patient study. The study was considered exempt and did not require consents since all applicable information had already been collected for clinical indication. A separate approval from the same Institutional Review Board was obtained to study normal subjects by carotid IMT but this approval did not include any blood draws. For the normal group, the approval required that informed consent was obtained.

### 2.1. Study Population

Our population consisted of pediatric patients (ages 5–20 yrs) initially evaluated between February 2008 and May 2010 who had CIMT measured in a standardized manner for clinical indication and who had no therapeutic lifestyle changes or pharmaceutical intervention prior to the visit. Records were randomly chosen retrospectively for MS patients, but an active search was required to identify individuals with heFH and MS+heFH. The control group consisted of 84 normal body habitus (no central overweight) volunteers who answered our advertisements in schools for subjects with no evidence of overweight and ages between 5 and 20 years. These subjects were imaged in accordance with a standardized IRB approved protocol similar to that used in prior pediatric carotid IMT studies [11–19].

### 2.2. Diagnostic Criteria

Patients diagnosed as having MS were required at presentation to have at least 3 of the 5 following findings on a gender specific basis: (1) systolic or diastolic blood pressure > 90th percentile of normal for age [20], (2) waist circumference > 90th percentile of normal for age [21], (3) triglycerides > 150 mg/dL, (4) HDL < 20th percentile of normal for age [22], and (5) evidence of fasting hyperglycemia > 100 mg/dL, or evidence of fasting hyperinsulinemia > the upper limit of normal, or definite acanthosis nigricans suggesting hyperinsulinemia. Diagnosis of heFH required evidence of the Simon-Broome criteria [23] except that we did not require evidence of tendinous xanthoma in family members since xanthomas are prevented by early treatment and somewhat uncommon in young family members. Fasting pretreatment lipid profiles were obtained prior to evaluation for the MS and heFH groups. Laboratory data were not available for the control group.

### 2.3. CIMT Measurement

CIMT measurements were performed with the same ultrasonic machine by ultrasonographers with >5 years of prior experience in CIMT recording. Reproducibility of the radio-frequency analysis technique (RFT) method for these technicians had been tested by repeatedly imaging 20 CIMT measurements 10 times at the same examination. Mean maximal difference between measurements was 3.0% or 12 *μ*m.

All studies were conducted using a Biosound Esaote MyLab30CV to record the CIMT ultrasounds. A 10–12-megahertz LA523 linear transducer was used for imaging the far wall of the right and left common carotid artery of each study participant. The posterior wall of CCA was insonated until a high quality image of the CIMT interfaces was obtained. This ultrasonic device employs real-time measurement made internally by the machine during the ultrasound examination to measure the far wall CIMT. Details and validation of RFT methodology are published elsewhere [24, 25]. A segment of the CCA was interrogated which stretched proximally from the area before widening of the carotid bulb back approximately 15 mm but avoided the first 1-2 mm proximal to the bulb. In a separate set of patients, CIMT interrogation was conducted from the carotid bulb to exactly 1 cm proximal to the bulb to determine if any statistical difference in CIMT was present between these two slightly different measurement techniques. Optimization of the CIMT measurement was conducted by adjusting the transducer angle to obtain the smallest standard deviation of RFT over several cardiac cycles. The output of the machine determined the mean and standard deviation for the length of the vessel that was evaluated for CIMT. Each CIMT was measured 3 times and the mean of these three measurements was reported.

### 2.4. Statistical Analysis

R and L CIMT were recorded for each patient included in the study. Group mean CIMT and standard deviation were calculated for each of the 4 groups in the study. The group mean CIMTs were compared with each other, as well as with the control group. The characteristics of each subgroup, such as age, LDL, HDL, non-HDL, and triglycerides, were analyzed by calculating means for these variables. To assess the statistical significance of the CIMT mean differences between individual groups as well as the lipid variables, we did a series of unpaired *t*-tests. An ANOVA test was considered to compare the 4 group means but was not used as substantial sample size differences, particularly for the heFH+MS group, would distort ANOVA results. A Pearson correlation coefficient was calculated for multiple potential risk factors, and multiple linear regression was used to determine lines of best fit and confidence intervals for each of the included risk factors in relation to CIMT. For a comparison of the two interrogation techniques, a paired *t*-test of the measurements of CIMT was used to determine if the techniques yielded statistically significant different results.

## 3. Results 

Data were analyzed for 90 children and teenagers aged 5–20 years with MS, heFH, or both heFH and MS. Of the 90, 57 were patients with MS, 23 with heFH, and 10 with heFH+MS. A separate group of 25 patients of the same age range were studied to determine if there was a significant difference in the two CIMT interrogation methods. The normal control group consisted of 84 normal, healthy volunteers with normal body habitus (no observable central overweight) and of the same age range. Mean BMI of the normal group was 19.3 (SD = 4.1). The mean age of all participants with lipid disorders was 13.5 (SD = 3.2). For the total heFH group (*n* = 33), 29 had family member(s) with Fredrickson type 11a familial hyperlipidemia and all had a first degree relative with a pretreatment LDL-C > 230 mg/dL and normal triglycerides, 2 were adopted with no available family history, and 2 had no known family member with Fredrickson type 11a and could have represented a new mutation or an autosomal recessive hypercholesterolemia. The mean age for the MS subgroup was 14 (SD = 2.9), heFH was 12.5 (SD = 3.7), MS+heFH was 12.7 (SD = 3.1), and the control group was 12.2 (SD = 4.2). The mean age of the participants with lipid disorders differed significantly from the mean age of the control group by 1.3 years (*P* = 0.031). The mean age of the MS subgroup differed from the control by 1.8 years (*P* = 0.004); the other subgroups did not differ significantly from the control group (Tables 1(a) and 1(b)).

### 3.1. Characteristics of the Metabolic Syndrome Group

The mean number of risk factors (maximum possible = 5) for the group with metabolic syndrome was 3.47 according to the criteria we utilized for diagnosis. Although complete data were available for blood pressure, waist, triglycerides, and HDL, we lacked fasting glucose in 20 patients. If we had adopted the International Diabetes Federation (IDF) criteria for metabolic syndrome [26], the mean number of risk factors would have dropped to 3.38 because of the lack of a fasting glucose. Only 7 patients had systolic hypertension by the IDF criteria, 3 had diastolic hypertension, and 2/3 with diastolic hypertension also had systolic hypertension. 78% had elevated triglycerides, 88% had acanthosis nigricans, 78% had low HDL (<40 mg/dL), 13% had fasting glucose > 100 mg/dL, and 96% had increased waist size.

### 3.2. Comparison of CIMT in the Groups

Comparison of two techniques of CIMT interrogation: the technique used to measure CIMT in our control group and comparison groups avoided the 2 mm of the CCA nearest to the carotid bulb. While, in older populations, the carotid bulb is the region most likely to have focal plaque deposition resulting in dissimilar CIMT measurements to other areas of the CCA, none of the patients included in our study had focal plaque deposition in this area. To mitigate any potential error posed by our chosen region of measurement, we compared interrogation including the 2 mm prior to the carotid bulb and the technique employed in our study (avoiding those 2 mm) and found a mean difference of 12 *μ*m, which was not statistically significant (*P* = 0.22).

**Figure 1:**
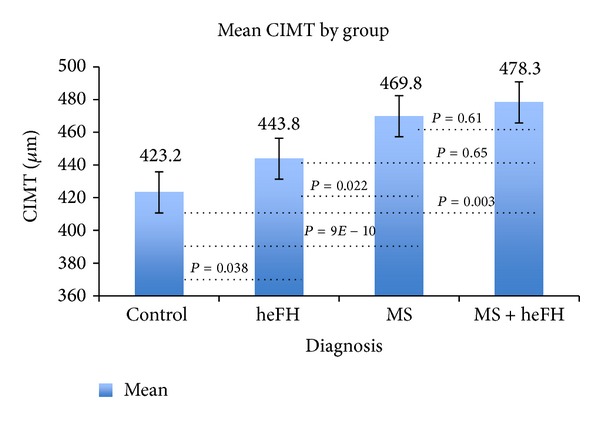
Mean CIMT is plotted for the control and disease categories.* P* values are shown comparing the several disease categories and the control. One SD is indicated by a single bar.

Mean CIMT was 469.8 *μ*m (SD = 67) for the MS group, 443.8 *μ*m (SD = 61) for the heFH group, 478.3 *μ*m (SD = 70) for the MS+heFH group, and 423.2 *μ*m (SD = 45) for the control group. Significance differences between groups were assessed with a series of unpaired *t*-tests: heFH versus MS (*P* = 0.022), heFH versus MS+heFH (*P* = 0.65), heFH versus control (*P* = 0.038), MS versus MS+heFH (*P* = 0.61), MS versus control (*P* = 9.0*E* − 10), and MS+heFH versus control (*P* = 0.003) (Figure 1 and Tables 1(a) and 1(b)).

### 3.3. Correlation and Multiple Linear Regression Analysis

**Figure 2:**
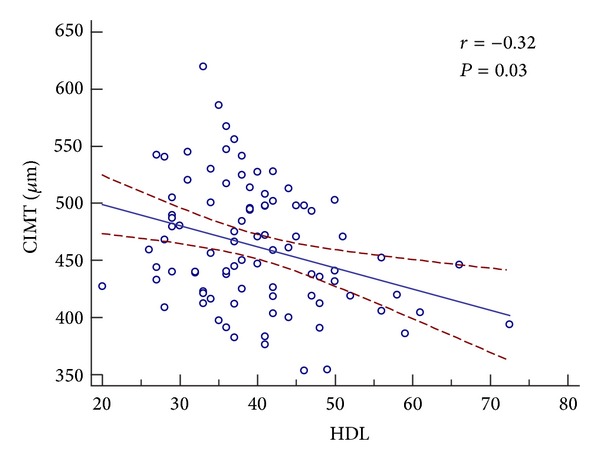
CIMT in *μ*m for each individual is plotted against HDL (mg/dL). The regression line is shown as solid line and the 95% confidence interval of the regression is plotted as the dashed line.

Pearson correlation coefficients and multiple linear regression analysis were used to determine correlation, lines of best fit, and confidence interval between several independent variables and CIMT. Significant negative correlation between HDL and CIMT was found (*r* = − 0.332,   *P* = 0.03). The other measured variables including LDL, triglycerides, waist circumference, BMI, and non-HDL did not show statistically significant correlation with CIMT (Table 2 and Figure 2).

### 3.4. Lipid Differences between Groups. 

Analysis of the subgroups included calculation of the means of important lipid variables: HDL, LDL, triglycerides, non-HDL, and participant age. For the MS group, the mean LDL was 116, HDL was 37, triglycerides were 208, and non-HDL was 157.6. For the heFH group, mean LDL was 178.4, HDL was 45.6, triglycerides were 140, and non-HDL was 206.3. For the MS+heFH group, mean LDL was 189.4, HDL was 43.2, triglycerides were 225.3, and non-HDL was 209.7 (Table 3).

To compare the significance of the differences between the means, a series of unpaired *t*-tests were conducted. For the MS versus heFH comparison, all three of these lipid variables differed significantly: LDL (*P* = 1.6*E* − 8), HDL (*P* = 0.001), triglycerides (*P* = 0.013), and non-HDL (*P* = 1.2*E* − 5). For the MS versus MS+heFH groups, only LDL and non-HDL were significantly different: LDL (*P* = 0.0003), non-HDL (*P* = 9.2*E* − 5), HDL (*P* = 0.19), and triglycerides (*P* = 0.73). For the heFH versus MS+heFH comparison, none of the lipid variables differed significantly: LDL (*P* = 0.48), HDL (*P* = 0.61), non-HDL (*P* = 0.074), and triglycerides (*P* = 0.13).

## 4. Discussions

The most significant finding of this study is that the MS group had a higher mean CIMT than the heFH group. Moreover, all three of the study groups had a significantly higher CIMT than the control group. Another potentially important result is the significant negative correlation between HDL and CIMT. Equally pertinent to our cohort is the finding that other risk factor measures such as LDL, non-HDL triglycerides, waist circumference, and BMI did not significantly correlate with CIMT.

Comparison of our data to that of Wiegman et al. [19] shows that the CIMT difference between the respective control groups and the respective heFH groups was almost identical (22 *μ*m versus 20.6 *μ*m). However, Wiegman et al. had higher values for both groups than we did, perhaps as a result of the different ultrasonic measurement technique employed. Reinehr et al. [27] evaluated CIMT in pediatric patients with MS and also found increased CIMT, but that study had no control group. Comparison of their mean CIMT to that of this study is not possible since they used nonstandard CIMT measurement [7] and accepted only the highest CIMT value of 4 measurements, whereas we used the mean of 3 consecutive measurements for each carotid. Comparison of our data for the normal group to normals of Doyon et al. [28] showed that their median was approximately 20 *μ*m lower than our medians at age levels. This was probably due to a slightly different site of measurement, as Doyon et al. measured CIMT more proximal to the bulb than we did, and values are slightly lower in that area.

The mean difference between the MS and heFH group was 26 *μ*m, with the MS group having a significantly thicker CIMT. Was this difference due to the 1.3-year age difference between the groups? In a 2009 study, CIMT was found to increase by approximately 6 *μ*m per year [25] in an adult population, and our pediatric control group showed a 1.6 *μ*m per year increase. Wiegman et al. also in a pediatric control group found an increase of 1.0 *μ*m per year. Gradual age-related increase does not account for the mean difference between the MS and heFH groups, as it would require a minimum of 5-year age difference between the MS and heFH groups in the current study, but the recorded mean difference was only 1.3 years. The MS+heFH group had the highest mean CIMT, approximately 8.5 *μ*m larger than MS alone, but, due to the small number of patients in this group, the study did not have the statistical power to show a difference between these groups if one existed. Means of both the MS and heFH groups were significantly elevated relative to our control group, with the MS group 46.6 *μ*m greater and the heFH group 20.6 *μ*m greater.

The finding that the mean of MS group is significantly elevated above that of the control group and the heFH group suggests the importance in the age range studied of the effect of MS on early CIMT thickening. The JUPITER trial showed that increased hsCRP, a measurement of inflammation, was independently related to future cardiovascular events, even in patients with LDL of 130 or less [5]. The JUIPTER result means that inflammation is a factor in atherosclerosis. Patients with MS also may have elevated hsCRP since MS has an inflammatory component. However, our results clearly cannot be compared to those of the JUPITER trial since JUPITER was an outcome study in older adults, and our study demonstrated the very early CIMT effect of MS in a pediatric population.

The Pearson correlation and multiple linear regression analysis provided potentially important information about how certain risk factors/variables correlated to CIMT. Previous studies have clearly related elevated LDL, decreased HDL, elevated BP, BMI, and waist circumference in children to increased vascular changes and adverse adult cardiovascular outcomes [1, 3, 4]. In our population, the only risk factor, which had a significant correlation to increased CIMT, was HDL, which had a significant negative correlation with CIMT (*r* = −0.333, *P* = 0.012). This may be important as it suggests the need to initiate therapeutic lifestyle changes aimed at reducing risk factors in pediatric MS patients.

Analyzing characteristics of the subgroups helps to substantiate the findings and rule out other possible confounders. First, the ages of the participants in each subgroup did not differ significantly, and each subgroup had a mean age between 12 and 14 years. The overall mean age was 13.5. Next, the MS and heFH group differed significantly in terms of the lipid characteristics of the participants. The heFH group had a mean LDL 62.4 mg/dL higher than the MS group, a mean non-HDL 48.8 mg/dL higher than the MS group, a mean HDL 8.4 mg/dL higher than the MS group, and triglycerides 68 mg/dL lower than the MS group. This is expected based on the characteristics and diagnostic definitions of each group. None of the lipid characteristics differed significantly when the heFH versus MS + heFH were compared. Another potential issue is whether our MS + heFH were true Fredrickson type 11a patients or whether they had familial combined hyperlipidemia. This can be a difficult differentiation without genetic testing. All 10 patients in this category had central overweight, criteria for MS, and 9/10 had an immediate family member with heFH, and that member had no evidence of metabolic syndrome and had normal triglycerides. Accordingly, the most likely diagnosis for this group was heFH + MS.

We tested two slightly different CIMT interrogation techniques as defined in the methods section. The reason for doing this was that most individuals have a mild widening of the common carotid artery just proximal to the bulb. The classic technique is to measure the posterior wall of the CCA CIMT in the 1 cm area prior to the bulb, but this can be subjective and the interrogation angle is not completely perpendicular as the common carotid artery widens. Nonetheless, this is an area where atherosclerotic changes occur in older patients. We demonstrated that both techniques provide a similar CIMT value in patients in the age range evaluated in this study by interrogating approximately 2 mm proximal to the bulb. The latter location is technically easier and more reproducible to measure. In adults with atherosclerosis, such a measurement technique change would probably not be advisable, due to the frequency of plaque deposition at this excluded site.

The current study has limitations. (1) The patient sample size of our study (*N* = 90) was relatively small. Only 10 individuals could be identified with both MS and heFH, making it difficult to have sufficient statistical power to show significant differences, if any existed, between this group and the other groups. A larger sample size may have been able to better define the differences, but individuals with both MS and heFH who have had no intervention are uncommon. (2) Our clinical laboratory data for the patients was that ordered by the referring physician rather than our choice of studies. Accordingly, we did not always have fasting glucose, insulin levels, or hsCRP during the initial evaluation when CIMT was measured. Use of the referring physician data was necessary since counseling at the initial visit might have altered the laboratory data when the patient obtained a second set. (3) This was a retrospective study involving data accumulated over two years. The effect of this limitation is minimized because the CIMT protocol of our laboratory was unchanged and performed in a standardized manner during that time. (4) We did not have lipid or glucose values for our normal control group. Although it is unlikely, we cannot rule out the possibility that one or more of our control group had familial hyperlipidemia or metabolic syndrome.

## 5. Conclusions

We conclude that the MS, heFH, and MS + heFH groups all have increased CIMT compared to our reference range control group and that the CIMT mean for the MS group is significantly elevated above the CIMT mean of the heFH group. Pediatric patients with MS have a mean CIMT approximately 46.6 *μ*m thicker than the control group and a CIMT mean 26 *μ*m thicker than the heFH group, and both are significant. HDL had a significant negative correlation with CIMT. Our findings indicate that pediatric patients with MS have significantly more thickening of CIMT than those with elevated LDL and non-HDL levels secondary to heFH.

## Figures and Tables

**Table:** (a)

Characteristic	MS	heFH	MS+heFH	Control
Age (years) (SD)	14.0 (±2.9)	12.5 (±3.7)	12.7 (±3.1)	12.2 (±4.2)
BMI (SD)	31.8 (±5.1)	23.4 (±4.8)	31 (±6.4)	19.3 (±4.1)
Waist circumference (cm) (SD)	104 (±13.4)	79.3 (±15)	97.3 (±15.7)	—
Blood pressure (mm/Hg)	122/74	112.8/69.6	111.5/71.7	—
CIMT (µm) (SD)	**469.8 (**±67)	**443.8 (**±61)	**478.3 (**±70)	**423.2 (**±45)
LDL (mg/dL) (SD)	116 (±25.3)	178.3 (±34.9)	189.4 (±42.4)	—
HDL (mg/dL) (SD)	37 (±6.9)	45.6 (±9.5)	43.2 (±136.1)	—
Triglycerides (mg/dL) (SD)	208 (±70.8)	139.8 (±112.3)	225.3 (±148.1)	—
Non-HDL (mg/dL)	157.6	206.3	209.7	—

**Table:** (b)

Comparisons	CIMT	LDL	HDL	TGs	Non-HDL	Age
MS versus heFH	0.022	1.6*E* − 08	0.0005	0.013	1.2*E* − 5	0.06
MS versus MS+heFH	0.61	0.0003	0.19	0.73	9.2*E* − 5	0.19
heFH versus MS+heFH	0.65	0.483	0.61	0.13	0.074	0.87
MS versus control	9*E* − 10	—	—	—	—	0.0035
heFH versus control	0.038	—	—	—	—	0.77
MS+heFH versus control	0.003	—	—	—	—	0.67

**Table 2:** Variables versus CIMT: multiple regression.

	*r*	*P*
LDL	0.15	0.71
HDL	**0.32**	**0.03**
Triglycerides	0.15	0.76
Non-HDL	0.08	0.44
Waist circumference	0.30	0.16
BMI	0.25	0.70

**Table 3:** Lipid values by group in mg/dL.

	MS	heFH	MS+heFH
LDL	116	178.4	189.4
HDL	37	45.6	43.2
Triglycerides	208	140.0	225.3
Non-HDL	158.6	206.3	209.7
